# Description of Hip Deformities in 5-Year-Old Patients with Congenital Zika Virus Syndrome: A Cross-Sectional Study

**DOI:** 10.1055/s-0044-1800922

**Published:** 2025-04-28

**Authors:** Herison Franklin Viana de Oliveira, Francisco Robson Queiroz Rego, Brauner de Souza Cavalcanti, Ítalo Rodrigues Bacellar, Thais Araújo Barbosa, Epitácio Leite Rolim Filho

**Affiliations:** 1 Departamento de Cirurgia, Universidade Federal de Pernambuco, Recife, PE, Brasil.; 2 Departamento de Ortopedia e Traumatologia, Hospital Getúlio Vargas, Recife, Pernambuco, PE, Brasil.; 3 Departamento de Ortopedia e Traumatologia, Universidade Federal de Pernambuco, Recife, PE, Brasil.

**Keywords:** child, deformities, developmental dysplasia of the hip, Zika virus

## Abstract

**Objective** To report on the most prevalent hip alterations in children older than 5 with congenital Zika virus syndrome (CZS) per clinical and radiographic examinations.

**Methods** Cross-sectional and retrospective study of 62 patients older than 5 with CZS. We extracted clinical data, including maximum abduction values, hip flexion contracture, gross motor function classification system (GMFCS) level, and radiographical data, including Reimers index (RI), femoral neck-shaft angle (FNSA), and acetabular index (AI), from medical records and radiographs and statistically evaluated them.

**Results** The mean age of children was 65.6 ± 4.1 months. Most patients (95.2%) presented GMFCS scores of IV and V. Slow hip abduction was 41.2 ± 19.5°. The Thomas test revealed a mean deviation of 16.1 ± 14.9°. The mean values of RI, FNSA, and AI were 54.1 ± 34.1%, 158 ± 11.9°, and 26.0 ± 8.12°, respectively. Patients with GMFCS III and IV had lower RI and AI than those with a score of V. Regarding FNSA, there was no statistical difference between groups. Patients who underwent tenotomy of the hip adductor muscles presented greater abduction but no relevant radiographic differences.

**Conclusion** There was a higher incidence of patients with hip luxation and more compromised functional degrees (GMFCS IV and V), and increased RI and AI in V. Operated patients presented abduction gain abduction but no radiographic improvement. Long-term studies are required to evaluate hip deformities in these subjects.

## Introduction

In 2015, Brazil faced an alarming increase in cases of microcephaly and a series of clinical alterations later known as the congenital Zika virus syndrome (CZS), 1 with 22,251 suspected and 3,742 confirmed cases by 2023. The infection persists, representing a continuous risk to pregnancy, and more than 2,000 affected children require multidisciplinary care and monitoring. 2

The neurological alterations from CZS increase muscle tone and spasticity, similar to upper motor neuron syndrome, leading to progressive musculoskeletal problems. 3 The hip is among the most affected joints, potentially presenting contractures, subluxations, and luxations resulting from muscle spasticity. These abnormalities cause debilitating functional effects, pain, and difficulties in hygiene, increasing the risk of infections and compromising quality of life. 4

Since the progression of deformity is fast, it is essential to maintain vigilance for early detection and immediate intervention. 5 Therapies for preventing hip luxation, similar to those used for spastic hips in cerebral palsy (CP), rely on physical and imaging examinations. 6 However, studies on the effectiveness of preventive measures in patients with CZS remain scarce.

Because of the lack of studies describing changes in hip development in CZS patients over time, the present aimed to inform the scientific community about the most prevalent changes in hips from children older than 5, correlating them with findings described in children with CP and using clinical and radiographic data.

## Materials and Methods

This cross-sectional, retrospective, descriptive study included children with CZS diagnosed by a neuropediatrician and followed up at the *Associação Pernambucana de Apoio à Criança com Deficiência* (AACD/PE) from August 2020 to January 2022. The study followed bioethical principles and received approval from the AACD/SP Research Ethics Committee (CAAE: 56150816.8.0000.0085).

This study included patients with CZS, born from June 2015 to May 2016, and older than 5 years of age during the research period. We excluded patients with ultrasound records of luxation at birth and multiple congenital joint contracture syndrome (arthrogryposis).

**Fig. 1:**
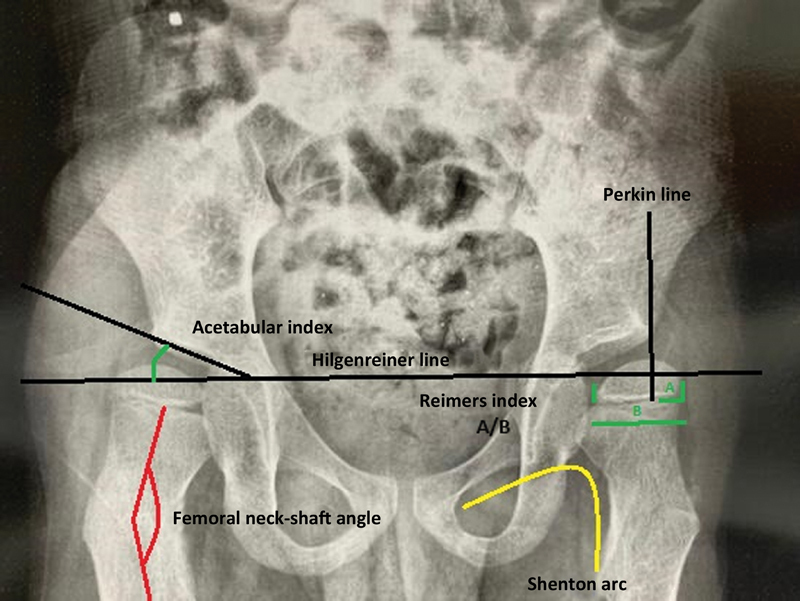
Anteroposterior pelvic radiograph with radiographic measurements.

We collected data from medical records containing clinical examinations. Two orthopedists, including one specialist in pediatric orthopedics, analyzed these children's radiographs and recorded the following values: maximum slow hip abduction with flexed knees and hip flexion contracture, per the Thomas test. We also assessed the patients' treatment, considering those undergoing tenotomy of the hip adductors as surgical and those undergoing physical therapy as nonsurgical. We recorded the Gross Motor Function Classification System (GMFCS) scores, and data from anteroposterior radiographs, including the acetabular index (AI; angles > 30° suggested dysplasia), femoral neck-shaft angle (FNSA), and Reimers index (RI; values > 90%, from 33 to 90%, and < 33% respectively indicated luxation, subluxation, and proper positioning), as shown in Fig. 1 .

For the statistical analysis, the categorical variables were expressed as frequencies and percentages, and the quantitative variables, as mean, standard deviation (SD), median, and interquartile range (IQR) values. The Shapiro-Wilk and Levene's tests for homogeneity of variances determined normality. The prevalence ratio compared categories based on nominal variables. We used the following tests for numerical variables: Student's *t* or Mann-Whitney tests for two categories, and analysis of variance (ANOVA) or nonparametric Kruskal-Wallis test for more than two categories. Spearman's correlation coefficient was used to perform an inductive analysis of dependent variables with nonnormal distribution. The significance level to reject or accept the null hypothesis was 5%.

## Results

Table 1 presents the demographic data of 62 children included in this study.

**Table 1:** Sample characterization

Variable	n	%
Children	62	100
Age (months) a	65.6 ± 4.1	–
Gender		
Male	28	45.2
Female	34	54.8
Gestational age at infection (weeks) a	14.07 ± 6.89	
Head circumference at birth a	28.57 ± 2.13	–
Gross motor function classification system		
I	0	–
II	0	–
III	3	4.8
IV	26	42.0
V	33	53.2
Treatment method		
Physical therapy, abduction orthosis, and/or botulinum toxin	45	72.6
Adductor tenotomy	17	27.4
Age at surgery (months) a	28.41 ± 9.04	
Hip status	n (124)	
Proper positioning	52	41.9
Subluxation	35	28.2
Luxation	37	29.9

**Note:**^a^ Mean ± standard deviation.

Most patients did not undergo surgical treatment until age 5, corresponding to 72.59% of the sample. Regarding the positioning status of the 124 hips evaluated, 52 (41.93%) were proper, as shown in Table 1 .

The physical examination revealed a mean slow hip abduction of approximately 41.2 ± 19.5°, and a quarter of the sample presented < 30° abduction. The Thomas test showed a mean value of 16.1 ± 14.9° with distribution between the first and third interquartile ranges. The mean RI, FNSA, and AI were 54.1 ± 34.1%, 158 ± 11.9°, and 26.0 ± 8.12°, respectively ( Table 2 ).

**Table 2:** Clinical and radiographic measurements

	ABD (degrees)	Thomas test (degrees)	RI (%)	FNSA (degrees)	AI (degrees)
N	124	124	124	108	112
Mean	41.2	16.1	54.1	158	26.0
Median	40.0	15.0	45.0	160	24.0
Standard deviation	19.5	14.9	34.1	11.9	8.12
25thpercentile	30.0	0.00	26.5	150	20.0
75thpercentile	50.0	25.0	92.0	168	32.0

**Abbreviations:** ABD, abduction; AI, acetabular index; FNSA, femoral neck-shaft angle; RI, Reimers index.

**Fig. 2:**
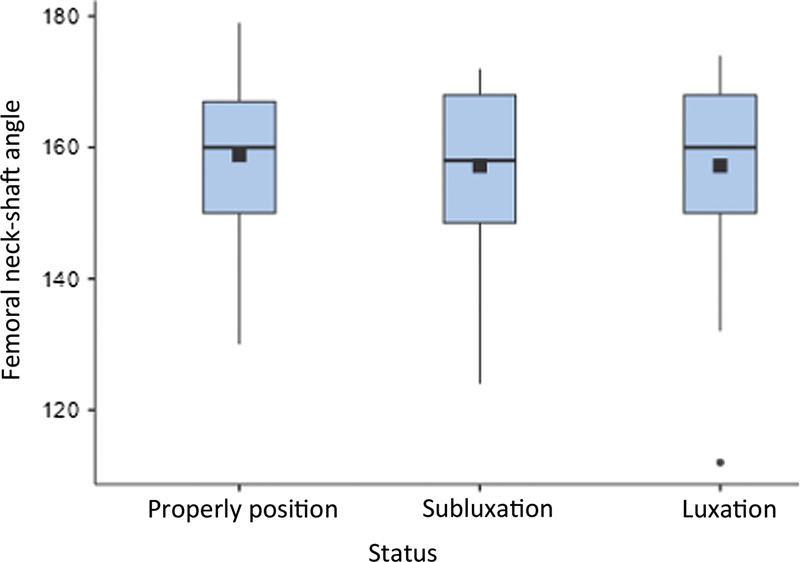
Femoral neck-shaft angle (degrees) versus hip status.

The FNSA and AI analysis relied on the deformity present (subluxation or luxation). There was no statistically significant correlation between FNSA and the deformities. However, AI was lower in properly positioned hips than in dislocated (subluxated and luxated) hips, as shown in Table 3 and Figs. 2 3 .

**Table 3:** Femoral neck-shaft angle and acetabular index relationship with the hip status

		Proper positioning	Subluxation	Luxation
**Femoral neck-shaft angle (degrees)**				
Proper positioning	Difference between mean values	—	1.670	1.654
	*p* -value	—	0.909 a	0.945 a
Subluxation	Difference between mean values		—	−0.012
	*p* -value		—	0.987 a
Luxation	Difference between mean values			—
	*p* -value			—
**Acetabular Index (degrees)**				
Proper positioning	Difference between mean values	—	−9.25	−11.21
	*p* -value	—	< 0.001 b,*	< 0 .001 b,*
Subluxation	Difference between mean values		—	−1.96
	*p* -value		—	0.528 b
Luxation	Difference between mean values			—
	*p* -value			—

**Notes:**^a^ Per the Kruskal-Wallis test with paired comparison by Dwass-Stell-Critchlow-Fligner. ^b^ Per the analysis of variance test with Welch correction and Games-Howell post-hoc test. *Significant different at the 5% level.

**Fig. 3:**
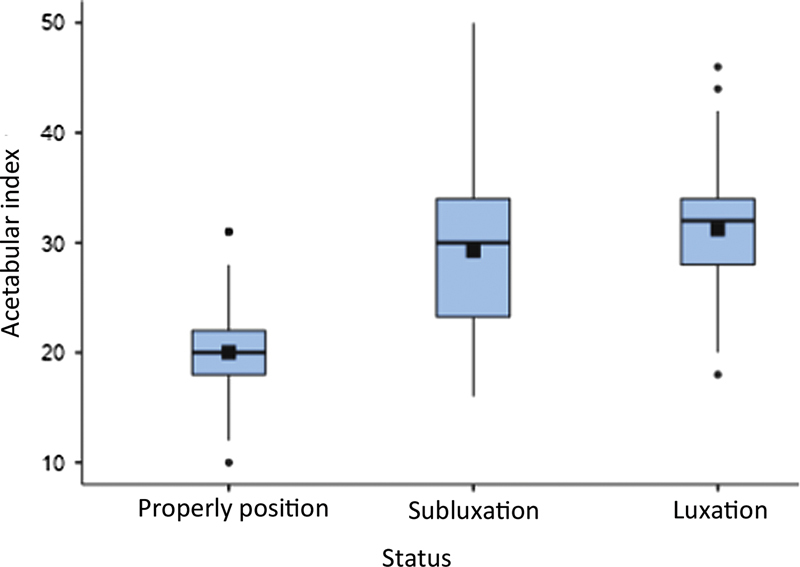
Acetabular index (degrees) versus hip status.

**Fig. 4:**
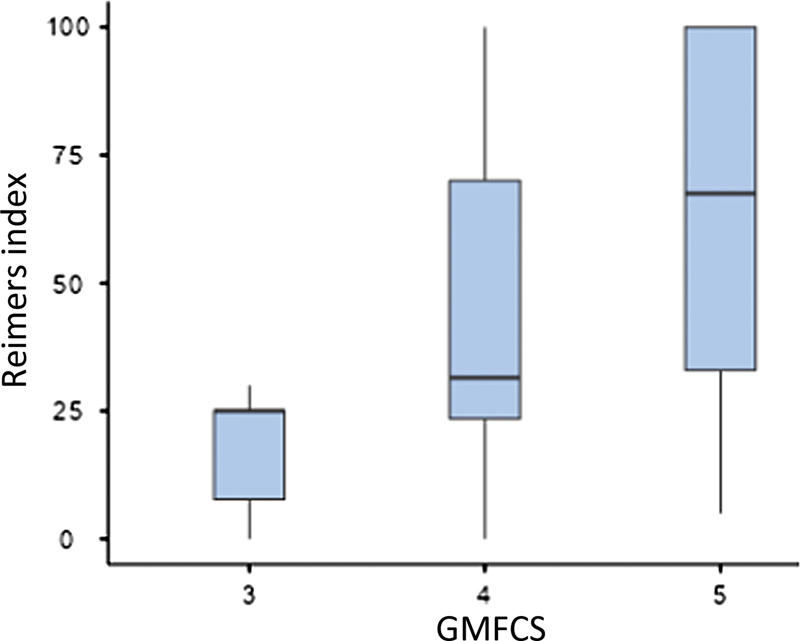
Reimers index versus gross motor function classification system (GMFCS).

Dividing the sample by their GMFCS score, we observed a statistical correlation in RI among patients from the three GMFCS groups. Considering hip subluxation (RI ≥ 33%) and GMFCS scores, hips with V had a subluxation prevalence 1.39-fold higher compared with IV ( *p* = 0.029; 95% confidence interval [CI]: 1.02–1.91). Patients with GMFCS III and IV had lower AI than those with V. There was no statistical difference between FNSA and the GMFCS groups ( Table 4 and Figs. 4 5 )

**Table 4:** Relationship of the RI, FNSA, and AI with the GMFCS

	GMFCS	N	Median	Percentile		MC	*p* -value a
				25th	75th		
RI	3	6	25.0	7.75	25.0	3 - 4	0.107
	4	52	31.5	23.50	70.0	3 - 5	0.003*
	5	66	67.5	33.00	100.0	4 - 5	0.011*
FNSA	3	6	146.5	145.25	153.0	3 - 4	0.052
	4	46	164.0	152.50	168.0	3 - 5	0.053
	5	56	160.0	150.00	168.0	4 - 5	0.790
AI	3	6	19.0	13.50	23.0	3 - 4	0.242
	4	46	22.0	18.00	30.0	3 - 5	0.014*
	5	60	28.5	22.00	34.0	4 - 5	0.008*

**Abbreviations:** AI, acetabular index; FNSA, femoral neck-shaft angle; GMFCS, gross motor function classification system; MC, multiple comparison; RI, Reimers index.

**Notes:**^a^ Per the Kruskal-Wallis test with paired comparison by Dwass-Stell-Critchlow-Fligner. *Significant different at the 5% level.

**Fig. 5:**
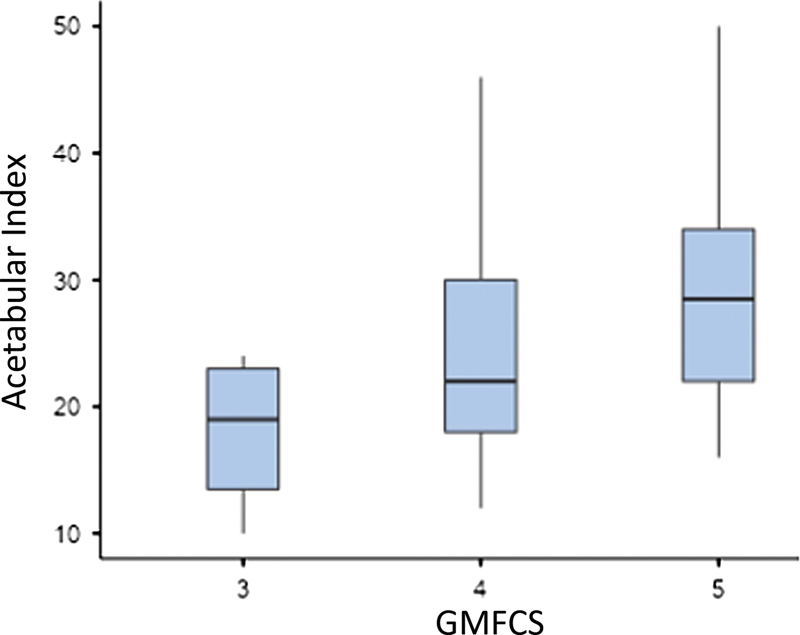
Acetabular index versus GMFCS.

There were 17 patients who underwent tenotomy of the hip adductor muscles (adductor longus, adductor brevis, and gracilis muscle) because they had RI > 33% and AI > 30°. Their mean age at surgery was 28.41 ± 9.04 months. These patients used an abduction orthosis for the first 2 months after the procedure and their mean follow-up time was 35.57 months. These patients presented no relevant difference in pre- and postoperative radiographic data. However, their physical examination indicated greater hip abduction among the operated patients.

Considering only surgically treated patients, we investigated GMFCS correlation with hip positioning, RI, AI, and FNSA but found no statistical correlation 2.96 years after surgery ( Table 5 ).

**Table 5:** Relationship between radiographic measurements and the type of treatment

	Treatment	N	Mean	Median	SD	Percentile		*p* -value ^a^
						25th	75th	
RI (%)	Surgical	34	58.8	55	34.35	27.3	100	0.825
	Non-surgical	90	52.3	38.5	34.06	25.0	90.8	
AI (degrees)	Surgical	24	29	30	5.97	24	34.0	0.992
	Non-surgical	88	25.2	24	8.46	20.0	30.3	
FNSA (degrees)	Surgical	20	157.7	162	15.39	151.5	168.0	0.680
	Non-surgical	88	157.9	160	11.09	150	168.0	
Hip abduction (degrees)	Surgical	34	47.6	45	19.5	30	67.5	0.018*
	Non-surgical	90	38.8	40	19	30	45	
Thomas test (degrees)	Surgical	34	14.6	15	12	2.5	20	0.348
	Non-surgical	90	16.6	15	15.9	0.5	25	

**Abbreviations:** AI, acetabular index; FNSA, femoral neck-shaft angle; RI, Reimers index; SD, standard deviation.

**Notes:**^a^ Per the Mann-Whitney test. *Significant different at the 5% level.

## Discussion

The CZS cases in Northeast Brazil accounted for more than half of those reported (53.1%), with the state of Pernambuco having the highest number. 2 The AACD-PE played an important role as a service provider for these patients. However, with the loss of follow-up over time, obtaining data 5 years after the initial contact was difficult.

The mean age of the 62 patients was 5.46 ± 0.34 years, a critical time frame since Wagner e Hägglund 7 reported that, at around 6-years-old, CP patients without previous surgeries present a tendency toward stabilization of the lateral migration velocity of the femoral head (increased RI). In contrast, at the same age, muscle contracture increases, potentially causing severe bone deformities. 8

Severe nervous system involvement and associated abnormalities impair neurodevelopment, preventing the acquisition of functional skills, as in CP. The GMFCS assesses a child's functional capacity and limitations, helping to determine motor prognosis and providing a better understanding of the severity of neurological impairment. 9 Ribeiro et al. 10 reported a prevalence of 88% of GMFCS IV and V in patients with CZS and a mean age of 13.9 months.

Similarly, Carvalho et al., 11 in 2019, evaluated children in the first year of life and reported a prevalence of 86.5%, and Cavalvante et al., 12 in 2021, followed children with the syndrome up to 36 months and reported a prevalence of 95% in the 110 patients with GMFCS IV and V. The present study corroborates this high prevalence by presenting a total of 59 patients (95.2%) with GMFCS IV and V. At the same time, Terjesen 13 investigated 335 children with CP and observed a prevalence of nondeambulatory children (GMFCS IV and V) of only 34%, which is consistent with Soo et al. 14 and Connely et al., 15 who reported 34 and 31%, respectively. This reflects the severity of neurological involvement and its influence on neurodevelopment in children with the syndrome.

Nervous system abnormalities, such as increased spasticity and delayed motor development, compromise hip development. Previous studies have described two mechanisms explaining the occurrence of hip deformities: muscle imbalance resulting from hip flexor and adductor spasticity outweighing the extensor and abductor muscles, increasing the force vector directing the femoral head away from the hip joint; and abductor muscle weakness due to delayed weight-bearing as determinants of the proximal femur anatomy. 16 17

In 2012, Terjesen 13 determined the velocity of lateral migration of the femoral head in children with CP but no CZS and demonstrated that complete hip dislocation occurred before age 6 in 11.3% of cases. Poirot et al. 18 reported that 53 of 218 hips (24.3%) of patients with CP had an RI > 40, and > 67% of them did not progress over 2.6 years. In the present study, 29.8% of the hips presented complete dislocation at age 5, raising the possibility of a more frequent unfavorable occurrence than in CP patients. The prevalence of proper positioning hips with RI < 33% was of 41.9%, contradicting Matos et al. 19 who reported 47 children with CZS and a prevalence of well-positioned hips of up to 90.4%. However, these authors did not include the age of the participants in their study.

As a monitoring recommendation, as in CP, hips with a maximum abduction of 30° and residual flexion in the Thomas test of 20° are at risk of developing subluxation or luxation. 5 In our sample, slow hip abduction with flexed knees had a mean angle of 41.2 ± 19.5°), and the Thomas test revealed a mean angle of 16.1 ± 14.9°. Excluding dislocated hips, 17 hips were considered at risk.

Despite a high FNSA, there was no statistical correlation between its increase and the presence or absence of subluxation or luxation. However, it is known that the presence of coxa valga alters the force vector acting on the hip, resulting in loss of joint congruence. 16 The lack of correlation in our study can result from age, as this interference is more prevalent in patients with CP older than 8. 20

Considering the ability to walk, Bobroff et al. 21 found a significantly elevated FNSA in 5-year-old patients who do not walk and a low FNSA among children with CP who do, which is consistent with observations from our sample regarding GMFCS IV and V patients compared to III, but without statistical significance. The increased FNSA in nondeambulatory subjects may result from the lack of vertical force in the proximal femur during development or a decreased traction force of the hip abductors on the greater trochanter. 21

Regarding AI, it was lower in properly positioned hips compared to subluxated or dislocated hips, with a difference between means of −9.25 ( *p* < 0.001) and −11.21° ( *p* < 0.001), respectively. This result is justified by the lack of adequate contact between the femur and acetabulum in the subluxation and luxation. 22 The GMFCS score also influenced AI since patients with V had a higher AI than those with III ( *p* = 0.014) and IV ( *p* = 0.008), consistent with Chung et al., 23 who studied spastic hips in patients without CZS.

In addition to more dysplastic hips (increased AI), children with GMFCS V also presented a higher tendency to lateralize the femur (increased RI) than those with lower motor levels. Aroojis et al. 16 reported in their study with spastic hips that the incidence of hip luxation (RI > 33%) was 2.5, 7.7, 50, 61, and 66% in GMFCS I, II, III, IV, and V, respectively. Barik et al., 24 in their systematic review, also observed an increase in lateralization velocity in CP patients with GMFCS of I–III, IV, and V by 0.3, 1.9, and 6.2% per year, respectively. In their study of patients with CP, Soo et al. 14 reported that the incidence of hip luxation was 69% in patients with GMFCS IV, with a relative risk 4.6 times higher for luxation than in those with II and worsening in V, with an incidence of 90% and a relative risk 5.9 times higher than in II. This corroborates the relationship found in our study, which shows a 1.39 times higher prevalence of hip luxation in children with GMFCS V compared to those with IV ( *p* = 0.029; 95% CI: 1.02–1.91). Likewise, the RI in patients with V was higher than in those with III ( *p* = 0.003) and IV ( *p* = 0.011).

To date, the treatment or prevention of hip deformities in children with CZS has followed the same indication patterns for children with CP, both surgical and nonsurgical. 25 Gordon and Simkiss 26 indicate surgery (tenotomy of the hip adductors) for patients with RI > 33% and AI > 30°. Ha et al. 27 considered a preoperative AI < 34° of the hip joints as an indicator of a better probability of success in preventing subluxation.

In the present study, all patients who underwent surgery had GMFCS IV or V. Although a study involving adductor tenotomy in 55 hips of children with CZS demonstrated a decrease in RI 12 months after the procedure, 28 patients who underwent surgical treatment did not present statistically significant improvements in radiographic measurements after virtually 3 years of surgery. While, by eliminating the force vector originated by the adductor muscles, operated subjects presented higher abduction ( *p* = 0.018) and less flexion contracture, there was no without statistical significance.

Regarding GMFCS, Shore et al. 29 observed progressively unfavorable tenotomy outcomes in patients with CP and abduction < 40° and RI > 30%, with success rates of 27 and 14% in subjects with IV and V, respectively. We attempted to perform the same correlation (GMFCS and RI) among our patients but found no statistical relationship ( *p* = 0.835).

## Conclusion

Children with CZS had a high incidence of hip dislocation and more severe neurological impairment (GMFCS IV and V). As in CP, children with GMFCS V had higher RI and AI, requiring greater surveillance. Surgery increases abduction capacity; however, studies with longer follow-ups are needed to address the natural history of hip deformities and the outcome of treatments performed in these patients.
