# Supplementary material for: Description of Hip Deformities in 5-Year-Old Patients with Congenital Zika Virus Syndrome: A Cross-Sectional Study
Source: Rev Bras Ortop (Sao Paulo). 2025 Apr 28;60(1):s00441800922. doi: 10.1055/s-0044-1800922 (PMC12037219; doi:10.1055/s-0044-1800922)
Supplement: Related texts

# Research article: Descrição das deformidades do quadril em pacientes com síndrome congênita do Zika vírus aos 5 anos de idade: Um estudo transversal

**Keywords:** criança, deformidades, displasia do desenvolvimento do quadril, Zika vírus

## Abstract

**Objetivo** Informar as alterações mais prevalentes no quadril em crianças com síndrome congênita do Zika vírus (SCZ) acima de 5 anos de idade por meio de exames clínicos e radiográficos.

**Métodos** Estudo transversal e retrospectivo em 62 pacientes com SCZ, com mais de 5 anos de idade durante o estudo. Os dados clínicos como valores de abdução máxima, contratura em flexão do quadril e nível do sistema de classificação da função motora grossa (SCFMG), assim como os dados radiográficos índice de Reimers (IR), ângulo colodiafisário (ACD) e índice acetabular (IA), foram extraídos de prontuários e radiografias e avaliados estatisticamente.

**Resultados** Idade média de 65,6 ± 4,1 meses. Prevalência de SCFMG IV e V foi 95,2%. A abdução lenta do quadril foi 41,2 ± 19,5°. O teste de Thomas revelou média de 16,1 ± 14,9°. Os valores médios do IR, ACD e IA, foram 54,1 ± 34,1%, 158 ± 11,9° e 26,0 ± 8,12°, respectivamente. Pacientes com SCFMG III e IV apresentaram IR e IA menores do que aqueles com V. Quanto ao ACD, não foi observada diferença estatística entre os grupos. Pacientes submetidos à tenotomia dos músculos adutores do quadril apresentaram abdução maior, mas sem diferença radiográfica relevante.

**Conclusão** Observa-se maior incidência de pacientes com luxação dos quadris e graus funcionais mais comprometidos (SCFMG IV e V), aumento do IR e IA no V. Pacientes operados tiveram ganho de abdução, porém sem melhora radiográfica. Estudos de longo prazo são necessários para a real avaliação das deformidades do quadril.

## Introdução

Em 2015, o Brasil enfrentou um aumento alarmante de casos de microcefalia e uma série de alterações clínicas mais tarde conhecidas como síndrome congênita do vírus Zika (SCZ), 1 com 22.251 casos suspeitos e 3.742 confirmados até 2023. A infecção persiste, representando um risco contínuo para a gravidez, e mais de 2.000 crianças afetadas necessitam de cuidados e acompanhamento multidisciplinar. 2

As alterações neurológicas da SCZ resultam em aumento do tônus muscular e espasticidade, semelhantes à síndrome do neurônio motor superior, levando a problemas musculoesqueléticos progressivos. 3 O quadril é uma das articulações mais afetadas, podendo sofrer contraturas, subluxações e luxações devido à espasticidade muscular. Essas abnormalidades causam efeitos funcionais debilitantes, dor e dificuldades na higiene, aumentando o risco de infecções e comprometendo a qualidade de vida. 4

Devido à rápida progressão das deformidades, é essencial manter a vigilância para detecção precoce e intervenção imediata. 5 Terapias para prevenção da luxação do quadril, semelhantes às usadas para o quadril espástico na paralisia cerebral (PC), são aplicadas com base em exames físicos e de imagem, 6 mas estudos sobre a eficácia das medidas preventivas na população afetada pela SCZ ainda são raros.

Devido à escassez de estudos que descrevam alterações no desenvolvimento do quadril de pacientes com SCZ ao longo do tempo, o presente estudo teve como objetivo informar a comunidade científica sobre as alterações mais prevalentes no quadril em crianças a partir de 5 anos de vida, correlacionando-as com os achados descritos em crianças com PC, utilizando dados clínicos e radiográficos.

## Materiais e Métodos

Este estudo transversal, retrospectivo e descritivo incluiu crianças com SCZ diagnósticas por neuropediatra e acompanhadas na Associação Pernambucana de Apoio à Criança com Deficiência (AACD/PE) entre agosto de 2020 e janeiro de 2022. Todo o estudo seguiu os princípios bioéticos e foi aprovado pelo Comitê de Ética e Pesquisa da AACD/SP. (CAAE: 56150816.8.0000.0085).

Foram incluídos pacientes com SCZ nascidos entre junho de 2015 e maio de 2016 e com idade superior a 5 anos de idade no período do estudo. Foram excluídos do estudo pacientes com registro ultrassonográfico de luxação ao nascimento e com síndrome de múltiplas contraturas articulares congênitas (artrogripose).

Os dados foram coletados de prontuários, contendo exame clínico e análise de exames radiográficos dessas crianças, realizados por dois ortopedistas, sendo um com especialização em ortopedia pediátrica. Foram contabilizados os valores de abdução lenta máxima do quadril, realizada com os joelhos flexionados, a contratura em flexão do quadril pelo teste de Thomas. Formas de tratamento aos quais foram submetidos, sendo cirúrgico aqueles submetidos à tenotomia dos adutores do quadril e não cirúrgico aqueles em acompanhamento fisioterápico, e ainda a avaliação dos níveis no sistema de classificação da função motora grossa (SCFMG); também foram registrados dados de exames radiográficos em incidência anteroposterior, tais como: índice acetabular (IA) com ângulos > 30° sugerindo existência de displasia, ângulo colodiafisário (ÂCD) e índice de Reimers (IR, com luxado > 90%; subluxado 33–90%; bem posicionado < 33%), como mostrado na Fig. 1 .

**Fig. 1:** Radiografia anteroposterior da pelve com mensurações radiográficas.

Análise estatística: as variáveis categóricas foram expressas como frequências absolutas e percentuais e as quantitativas, como médias, desvios padrão (DP), medianas e intervalos interquartis (IIQ). Para verificar a normalidade foi utilizado o teste de Shapiro-Wilk e de Levene para homogeneidade de variâncias. A razão de prevalência foi utilizada para comparação entre categorias com base em variáveis nominais, enquanto os seguintes testes foram utilizados para variáveis numéricas: os testes de Mann-Whitney ou *t* de Student para duas categorias e análise de variância (ANOVA) ou teste não paramétrico de Kruskal-Wallis para mais do que duas categorias. Para análise indutiva das variáveis dependentes com distribuição não normal foi utilizado o coeficiente de correlação de Spearman. O nível de significância utilizado para rejeitar ou aceitar a hipótese nula foi de 5%.

## Resultados

Os dados demográficos de 62 crianças deste estudo são apresentados na Tabela 1 .

**Tabela 1:** Caracterização da amostragem

Variável	n	%
Crianças	62	100
Idade (meses) a	65,6 ± 4,1	–
Gênero		
Masculino	28	45,2
Feminino	34	54,8
Idade gestacional na infecção (semanas) a	14,07 ± 6,89	
Perímetro encefálico ao nascimento a	28,57 ± 2,13	–
Sistema de classificação da função motora grossa		
I	0	–
II	0	–
III	3	4,8
IV	26	42,0
V	33	53,2
Método de tratamento		
Fisioterapia, órtese de abdução e/ou toxina botulínica	45	72,6
Tenotomia dos adutores	17	27,4
Idade na cirurgia (meses) a	28,41 ± 9,04	
Situação dos quadris	124	
Locados	52	41,9
Subluxados	35	28,2
Luxados	37	29,9

**Nota:**^a^ Média ± desvio padrão.

A maioria dos pacientes não realizou tratamento cirúrgico até os 5 anos de idade, correspondendo a 72,59% da amostra. Quanto à situação de posicionamento dos 124 quadris avaliados, 52 (41,93%) encontravam-se locados, visto na Tabela 1 .

Ao exame físico, a abdução lenta média do quadril foi de aproximadamente 41,2 ± 19,5°, sendo que um quarto da amostra apresentou abdução < 30°. O teste de Thomas teve média de 16,1 ± 14,9° com distribuição entre o 1 e 3° interquartis. Foram registrados IR, ACD e IA, com valores médios de 54,1 ± 34,1%, 158 ± 11,9° e 26,0 ± 8,12°, respectivamente ( Tabela 2 ).

**Tabela 2:** Medidas clínicas e radiográficas

	ABD (graus)	Teste de Thomas (graus)	IR (%)	ÂCD (graus)	IA (graus)
N	124	124	124	108	112
Média	41.2	16.1	54.1	158	26.0
Mediana	40.0	15.0	45.0	160	24.0
DP	19.5	14.9	34.1	11.9	8.12
25° percentil	30.0	0.00	26.5	150	20.0
75° percentil	50.0	25.0	92.0	168	32.0

**Abreviações:** ABD, abdução; DP, desvio padrão; ACD, ângulo colodiafisário; IA, índice acetabular; IR, índice de Reimers.

O ACD e o IA foram analisados conforme a deformidade presente (subluxação ou luxação). Não foi observada correlação estatisticamente significativa entre o ACD e as deformidades. Entretanto, considerando o IA, esse ângulo foi reduzido ao comparar os quadris locados com os deslocados (subluxados e luxados), conforme apresentado na Tabela 3 e Figs. 2 3 .

**Tabela 3:** Relação do ângulo colodiafisário e do índice acetabular com as situações dos quadris

		Locado	Subluxado	Luxado
**Ângulo colodiafisário (graus)**				
Locado	Diferença entre as médias	—	1,670	1,654
	Valor de *p*	—	0,909 a	0,945 a
Subluxado	Diferença entre as médias		—	−0,012
	Valor de *p*		—	0.987 a
Luxado	Diferença entre as médias			—
	Valor de *p*			—
**Índice acetabular (graus)**				
Locado	Diferença entre as médias	—	−9.25	−11.21
	Valor de *p*	—	< 0.001 b,*	< 0 .001 b,*
Subluxado	Diferença entre as médias		—	−1.96
	Valor de *p*		—	0.528 b
Luxado	Diferença entre as médias			—
	Valor de *p*			—

**Notas:**^a^ Pelo teste Kruskal-Wallis com comparação entre pares por Dwass-Stell-Critchlow-Fligner. ^b^ Pela teste de análise de variância com correção de Welch e teste post hoc de Games-Howell. *Diferença significativa ao nível de 5%.

**Fig. 2:** Ângulo colodiafisário (graus) versus situação do quadril.

**Fig. 3:** Índice acetabular (graus) versus situação do quadril.

Observando a amostra dividida em grupos com base no SCFMG, foi verificada correlação estatística no IR nos pacientes entre os três grupos SCFMG. Considerando a presença de subluxação do quadril (IR ≥ 33%) com base no nível de SCFMG, os quadris de crianças com V apresentam prevalência de subluxação 1,39 vezes maior do que aquelas com IV ( *p* = 0,029; intervalo de confiança de 95% [IC]: 1,02–1,91). Pacientes com SCFMG III e IV apresentaram IA menor que aqueles com V. Quanto ao ACD, não foi observada diferença estatística entre os grupos ( Tabela 4 e Figs. 4 5 )

**Tabela 4:** Relação do IR, ACD e IA com o SCFMG

				Percentil			
	SCFMG	N	Mediana	25°	75°	CM	Valor de *p* a
IR	3	6	25.0	7.75	25.0	3 - 4	0,107
	4	52	31.5	23.50	70.0	3 - 5	0,003 *
	5	66	67.5	33.00	100.0	4 - 5	0,011 *
ACD	3	6	146.5	145.25	153.0	3 - 4	0,052
	4	46	164.0	152.50	168.0	3 - 5	0,053
	5	56	160.0	150.00	168.0	4 - 5	0,790
IA	3	6	19.0	13.50	23.0	3 - 4	0,242
	4	46	22.0	18.00	30.0	3 - 5	0,014 *
	5	60	28.5	22.00	34.0	4 - 5	0,008 *

**Abreviações:** ACD, ângulo colodiafisário; CM, comparação múltipla; IA, índice acetabular; IR, índice de Reimers; SCFMG, sistema de classificação da função motora grossa.

**Notas:**^a^ Pelo teste Kruskal-Wallis com comparação entre pares por Dwass-Stell-Critchlow-Fligner. *Diferença significativa ao nível de 5%.

**Fig. 4:** Índice de Reimers versus sistema de classificação da função motora grossa (SCFMG).

**Fig. 5:** Índice acetabular versus SCFMG.

Foram 17 pacientes submetidos à tenotomia dos músculos adutores do quadril (adutor longo, adutor curto e músculo grácil) por possuírem IR > 33% e IA > 30°. Tinham idade média de 28,41 ± 9,04 meses quando operaram, fizeram uso de órtese de abdução nos 2 primeiros meses pós-cirúrgico e tiveram tempo médio de seguimento de 35,57 meses. Esses pacientes não apresentaram diferença relevante nos dados radiográficos pré- e pós-operados. Entretanto, o exame físico indicou maior abdução do quadril entre os pacientes operados.

Considerando apenas os pacientes tratados cirurgicamente, foi investigada a correlação do SCFMG entre as variáveis posição, IR, IA e ACD; no entanto, nenhuma correlação estatística foi observada 2,96 anos após a cirurgia ( Tabela 5 ).

**Tabela 5:** Relação de medidas radiográficas com o tipo de tratamento

	Tratamento	N	Média	Med.	DP	Percentil		Valor de *p* ^a^
						25°	75°	
IR (%)	Cirúrgico	34	58,8	55	34,35	27,3	100	0,825
	Não cirúrgico	90	52,3	38,5	34,06	25,0	90,8	
IA (graus)	Cirúrgico	24	29	30	5,97	24	34.0	0,992
	Não cirúrgico	88	25,2	24	8,46	20.0	30.3	
ACD (graus)	Cirúrgico	20	157,7	162	15,39	151,5	168.0	0,680
	Não cirúrgico	88	157.9	160	11,09	150	168.0	
Abdução dos quadris (graus)	Cirúrgico	34	47,6	45	19,5	30	67,5	0,018*
	Não cirúrgico	90	38,8	40	19	30	45	
Teste de Thomas (graus)	Cirúrgico	34	14,6	15	12	2,5	20	0,348
	Não cirúrgico	90	16,6	15	15,9	0,5	25	

**Abreviações:** ACD, ângulo colodiafisário; DP, desvio padrão; IA, índice acetabular; IR, índice de Reimers, Med., mediana.

**Notas:**^a^ Pelo teste Mann-Whitney. *Diferença significativa ao nível de 5%.

## Discussão

Os casos de SCZ no Nordeste do Brasil respondem por mais da metade dos notificados (53,1%), com Pernambuco tendo o maior número de casos. 2 Coube à AACD-PE um importante papel como prestadora de serviços de atendimento a estes pacientes. Porém, com a perda de acompanhamento ao longo do tempo, a obtenção de dados 5 anos após o primeiro contato foi um desafio para a realização do estudo.

A média de idade dos 62 pacientes foi de 5,46 ± 0,34 anos, marco temporal importante visto que Wagner e Hägglund 7 relataram que, por volta dos 6 anos de idade, pode ser observada uma tendência à estabilização da velocidade de migração lateral da cabeça femoral (IR aumentado) em pacientes com PC sem cirurgia prévia. Por outro lado, na mesma idade a contratura muscular aumenta, o que pode causar graves deformidades ósseas. 8

O comprometimento grave do sistema nervoso bem como as alterações associadas causa prejuízo ao neurodesenvolvimento, impedindo a aquisição de habilidades funcionais, como na PC. O SCFMG é utilizado para avaliar a capacidade e limitações funcionais de uma criança, servindo como guia para determinar o prognóstico motor e ajuda a compreender melhor a gravidade do comprometimento neurológico. 9 Ribeiro et al. 10 encontraram prevalência de 88% de SCFMG IV e V em pacientes com SCZ com idade média de 13,9 meses.

Da mesma forma, Carvalho et al., 11 em 2019, avaliaram crianças no primeiro ano de vida e relataram prevalência de 86,5%, e Cavalvante et al., 12 em 2021, acompanharam crianças com a síndrome até 36 meses e apontaram prevalência de 95% dos 110 pacientes categorizados entre SCFMG IV e V. O presente estudo corrobora esta alta prevalência por apresentar um total de 59 (95,2%) pacientes, classificada como SCFMG IV e V. Paralelamente, Terjesen 13 investigou 335 crianças com PC e observou uma prevalência de crianças não deambuladoras (SCFMG IV e V) de apenas 34%, achado consistente com o dos trabalhos de Soo et al. 14 e Connely et al., 15 que relataram 34 e 31%, respectivamente. Isto reflete a gravidade do envolvimento neurológico e sua influência no neurodesenvolvimento nas crianças com a síndrome.

Alterações do sistema nervoso, como aumento da espasticidade e atraso no desenvolvimento motor, comprometem o desenvolvimento do quadril. Os estudos anteriores descreveram dois mecanismos que explicam a ocorrência de deformidades do quadril: desequilíbrio muscular gerado pela espasticidade dos flexores e adutores do quadril que superam os extensores e abdutores, aumentando um vetor de força que direciona a cabeça femoral para longe da articulação do quadril; e a fraqueza da musculatura abdutora associada ao atraso na sustentação de peso como determinantes da anatomia do fêmur proximal. 16 17

Em 2012, Terjesen 13 realizou um estudo determinando a velocidade de migração lateral da cabeça femoral em crianças com PC sem SCZ e demonstraram que a luxação completa do quadril ocorreu antes dos 6 anos de idade em 11,3% dos casos. Poirot et al. 18 relataram que 53 de 218 quadris (24,3%) de pacientes com PC tinham um IR > 40, e > 67% deles não progrediram ao longo de 2,6 anos. No presente estudo, 29,8% dos quadris apresentavam luxação completa aos 5 anos, levantando a possibilidade de ocorrência desfavorável mais frequente do que em pacientes com PC. A prevalência de quadris locados, ou seja, com IR < 33%, foi de 41,9%, contrariando o estudo de Matos et al. 19 com 47 crianças com SCZ, no qual a prevalência de quadris bem-posicionadas chegou a 90,4%. No entanto, a idade dos participantes não foi informada.

Como recomendação de vigilância foi considerado, assim como na PC, que quadris com abdução máxima de 30° e flexão residual no teste de Thomas de 20° correm risco de evoluir com subluxação ou luxação. 5 Nesta amostra, a abdução lenta do quadril com joelhos flexionados teve média de 41,2 ± 19,5° e o teste de Thomas, média de 16,1 ± 14,9°. Excluindo os quadris já luxados, 17 quadris foram considerados de risco.

Apesar de um ACD elevado, não foi observada correlação estatística entre o aumento do ACD com a presença ou ausência de subluxação ou luxação. No entanto, sabe-se que a presença de coxa valga altera o vetor de força que atua no quadril, resultando na perda de congruência articular. 16 A falta de correlação neste estudo pode ser explicada pela idade, pois essa interferência é mais prevalente após os 8 anos de idade em pacientes com PC. 20

Considerando a capacidade de caminhar, Bobroff et al. 21 encontraram um ACD significativamente elevado em pacientes de 5 anos de idade que não andam e um ACD baixo entre crianças com PC que andam, o que é consistente com o observado nesta amostra para pacientes SCFMG IV e V em comparação ao nível III, mas sem significado estatístico. O aumento do ângulo colodiafisário em não deambuladores pode ser explicado pela falta de força vertical no fêmur proximal durante o desenvolvimento ou pela diminuição da força de tração dos abdutores do quadril no trocânter maior. 21

Quanto ao IA, observa-se medida menor nos quadris locados em comparação aos quadris subluxados ou luxados, com diferença entre as médias de −9,25 ( *p* < 0,001) e −11,21° ( *p* < 0,001), respectivamente. Justificando-se pela falta de contato adequado entre fêmur e acetábulo nestes últimos. 22 O IA também é influenciado pelo nível do SCFMG, pois pacientes com nível V apresentaram IA maior do que aqueles com níveis III ( *p* = 0,014) e IV ( *p* = 0,008), consistente com o estudo de Chung et al. 23 que estudaram quadris espásticos sem Zika.

Além de quadris mais displásicos (IA aumentado), crianças com SCFMG V também apresentam maior tendência a lateralizar o fêmur (IR aumentado) em comparação com aquelas com níveis motores mais baixos. Aroojis et al. 16 relataram em seu estudo com quadris espásticos que a incidência de luxação do quadril (IR > 33%) foi de 2,5, 7,7, 50, 61 e 66% no SCFMG I, II, III, IV e V, respectivamente. Barik et al., 24 em sua revisão sistemática, também observaram um aumento na velocidade de lateralização em pacientes com PC com SCFMG de I–III, IV e V em 0,3, 1,9 e 6,2% por ano, respectivamente. Em seu estudo com pacientes com PC, Soo et al. 14 relataram que a incidência de luxação do quadril foi de 69% em pacientes com SCFMG IV, com risco relativo 4,6 vezes maior de luxação do que aqueles com II, piorando no V, com incidência de 90% e risco relativo 5,9 maior que aquele no II. Isso corrobora a relação encontrada neste estudo, mostrando prevalência 1,39 vezes maior de deslocamento do quadril em crianças com SCFMG V em comparação com aquelas com nível IV ( *p* = 0,029; IC95%: 1,02–1,91). Da mesma forma, o IR em pacientes com SCFMG V é maior que naqueles com III ( *p* = 0,003) e IV ( *p* = 0,011).

O tratamento ou prevenção de deformidades do quadril em crianças com SCZ seguiu, até o momento, os mesmos padrões de indicação utilizados para crianças com PC, tanto cirúrgico como não cirúrgico. 25 Assim como Gordon e Simkiss 26 a cirurgia (tenotomia dos adutores do quadril) foi indicada para pacientes com IR > 33% e IA > 30°. Ha et al. 27 considerou um IA pré-operatório < 34° da articulação do quadril um indicador de melhor probabilidade de sucesso na prevenção da subluxação.

No presente estudo, todos os pacientes operados apresentavam SCFMG IV ou V. Embora, um estudo envolvendo tenotomia dos adutores em 55 quadris de crianças com SCZ tenha demonstrado diminuição do IR 12 meses após o procedimento, 28 os pacientes submetidos a tratamento cirúrgico não apresentaram melhora estatisticamente significativas das medidas radiográficas após quase 3 anos da cirurgia. No entanto, devido a eliminação do vetor de força originado pelos músculos adutores, observou-se maior abdução entre os operados ( *p* = 0,018) e menor contratura em flexão, mas sem significado estatístico.

Comparando com o SCFMG, Shore et al. 29 observaram resultados progressivamente desfavoráveis para tenotomia realizada em pacientes com PC com abdução < 40° e IR > 30%, com sucesso de 27 e 14% em pacientes com níveis IV e V, respectivamente. Tentamos fazer a mesma correlação (SCFMG e IR) entre as crianças operadas neste estudo, mas não foi encontrada relação estatística ( *p* = 0,835).

## Conclusão

As crianças com SCZ apresentaram elevada incidência de luxação dos quadris e um maior comprometimento neurológico (SCFMG IV e V). Assim como na PC, as crianças com SCFMG V apresentaram maiores IR e IA, exigindo maior vigilância. A realização da cirurgia aumenta a capacidade de abdução; entretanto, fazem-se necessários estudos com maior tempo de seguimento abordando a história natural das deformidades nos quadris e o desfecho quanto aos tratamentos realizados nestes pacientes.
